# Improving the “global use” of ultrasound for central venous access: a new supraclavicular scan by microconvex probe

**DOI:** 10.1186/2036-7902-6-S2-A11

**Published:** 2014-08-27

**Authors:** A La Greca, DG Biasucci, A Emoli, M Pittiruti

**Affiliations:** 1 Emergency Surgery Unit, “A. Gemelli” Hospital, Catholic University, Roma, Italy; 2 Dept. of Anesthesia, “A. Gemelli” Hospital, Catholic University, Roma, Italy; 3 Dept. of Oncology, “A. Gemelli” Hospital, Catholic University, Roma, Italy

## Background

Ultrasound (US) is increasingly used as a “global” tool to assist all the steps of a central venous catheter insertion procedure: choice of vein/approach, needle guidance during venepucture, prevention of primary malposition, rule out of pleural complications. While approach choosing and needle guidance are now well standardized, US-based prevention of primary malposition and rule out of pleural complications are still a matter of debate.

US based prevention of primary malposition of a central catheter relies on [1-4]:

- rule out of catheter misdirection in the superior vena cava (SVC) tributary veins (so called “negative assessment”)

- identification of the tip within the right atrium and/or in the lower part of the SVC (so called “positive assessment”). Tip position may be evaluated:

○ by direct visualization of the tip within the cardiac chambers and/or the lower part of the SVC

○ indirectly by contrast enhanced ultrasound.

Prerequisite for a reliable negative and positive assessment is a good visualization of the entire central venous axis, including the brachiocefalic veins, the lower segment of the SVC and the right atrium.

Negative assessment is usually performed by scanning the internal jugular (IJ), axillary and brachiocefalic veins.

Different scanning techniques have been described for the positive assessment, in terms of:

- acoustic windows: anterior trans-thoracic or subcostal

- type of probes: medium (convex) or low (conic) frequency

Both negative and positive assessment have significant limitations.

- Negative assessment: a malposition in the contralateral subclavian/brachiocefalic vein may be underestimated because of a US-blind venous segment beneath the clavicule, especially on the left side

- Positive assessment: a different probe from the one used during the ultrasound-guided venepuncture is required, and even using high-quality devices, the acoustic windows are often suboptimal; moreover, a high level of training is required.

Rule out of pleural complications often requires a different (usually medium-low frequency) probe from the one used during the ultrasound-guided venepuncture.

## Objective

The end-point of the study was to to determine the feasibility of using the microconvex probe as a unique tool to manage the four main steps of a CVC insertion procedure: 1) choice of vein/approach, 2) safe US-guided venepuncture, 3) catheter direction and tip position control, 4) rule out of pleural complications. In particular, the hypothesis of managing step three by means of a right supraclavicular scan (which allows direct visualization of the entire brachiocephalic-caval venous axis to support US-based catheter direction and tip location control) was evaluated.

## Methods

The so-called microconvex probe is an intermediate medium-high frequency probe, which couples:

- the good image resolution of a high frequency (usually linear in shape) probe, required for venepuncture guidance;

- the scanning depth (up to 10-12 cm and more) of a medium-frequency (usually convex in shape) one, required for rule-out of pleural complications, i.e. pneumothorax and fluid collections.

Moreover, the microconvex probe has a convenient convex but small exploration surface, allowing it to be easily used in narrow anatomical regions, such as the intercostal spaces or the supraclavicular fossa [5,6].

This pilot study was designed in two steps .

### Step A

The microconvex probe was tested on 10 consecutive patients in order to define its accuracy in detecting three goal-images: .

1. Nerveo-vascular bundle (vein, artery, nerve) in three anatomical regions for each patient:

a. lower cervical (carotid artery – jugular vein – vagus nerve);

b. lateral subclavear (axillary vein with cephalic vein inlet, axillary artery);

c. medial brachial at the medium third (brachial veins + basilic, brachial arteries, median nerve).

These images are considered essential for applying the RaCeVA protocol and for subsequent US-guided venepuncture.

2. Brachiocefalic-caval venous axis seen by the supraclavicular approach. The microconvex probe is placed on the right supraclavicular fossa and directed towards the mediastinum to obtain a coronal scan of the entire brachiocefalic-caval venous axis + a transverse section of the aortic arch medial to the higher segment of the SVC + a longitudinal section of the right pulmonary artery crossing the SVC as a landmark of its lower third. **This image is primarily needed for integrating rule out of catheter misdirection and for tip position control.**

3. Gliding and sliding signs + B-Lines via a transthoracic longitudinal scan at the second/third intercostal space along the parasternal line omolateral to the venepuncture site. **This image is needed for ruling out pleural complications**.

### Step B

Afterwards, 6 consecutive patients underwent a CVC insertion procedure. Our standard insertion protocol is described elsewhere and includes a complete US examination of explorable cervico-thoracic veins (RaCeVA protocol) to choose the best approach in each patient, US-guided venepuncture, US-based rule out of catheter misdirection (“negative assessment”), intracavitary ECG controlled tip placement, US-based rule out of pleural complications using linear and convex probes. This standard protocol was modified introducing the microconvex probe to:

a. perform the RaCeVA protocol as usual;

b. guide the venepuncture with the same techniques usually coupled with a linear probe;

c. visualize the catheter/guidewire within the great vessels via the right supraclavicular scan according to the image parameters described in A2 in order to rule out catheter misdirection and locate the tip deeply in the caval axis;

d. rule out of pneumothorax and pleural collections without changing the exploring probe.

## Results

- Stage A. The goal images were clearly obtained in all patients.

- Stage B. US-guided venepuncture was successful in all patients. The probe allowed a good visualization of:

○ the needle in plane entering the vessel in short axis (postero-inferior approach to the internal jugular vein 3 cases, 2 from the right side and one from the left side); in one very difficult case (deep IJV, moving into the operative US field synchronously with respiration; no feasible alternative veins), the convex probe shape allowed a very good needle visualization even though the narrow space available

○ the needle out plane entering the vessel in short axis (subclavear approach to the axillary vein, 3 cases, 2 from the right side, 1 from the left side)

The entire course of the metal guidewire within the SVC was visible in all 6 patients, but the guidewire tip was clearly recognizable deeply at the crossing between the SVC and the right pulmonary artery in only 2 cases. The catheter was not recognizable within the great vessels, maybe because of its low echogenicity as compared to the metal guidewire and/or because of training issues.

No primary misdirectons of the guidewire wee recorded. No pleural complications were detected.

## Conclusion

The microconvex probe is a useful tool to manage all the steps of a CVC insertion procedure, including choice of vein/approach, US-guided venepuncture, with a special effectiveness in approaching difficult deep veins, and rule out of pleural complications without the need to change the probe at the end of the procedure to scan the pleural cavity. The supraclavicular scan is an uncommon but promising scanning view allowing visualization of the guidewire within the great vessels deep into the SVC (thus increasing the global accuracy in correctly direct the catheter), but its effectiveness in exactly locate the tip needs further research and/or training.
